# Developing a novel computer visualization system to simulate the uranium upward transport mechanism: Uranium pollution in arid landscapes

**DOI:** 10.1016/j.mex.2022.101794

**Published:** 2022-07-25

**Authors:** Joshua E. Lou, Lucas F. Larson, Samuel M. Han, Naira Ibrahimd, Fengxiang X. Han

**Affiliations:** a Oxford Academy, 5172 Orange Ave, Cypress, CA 90630; b St. Aloysius High School, Vicksburg, MS 39180; c Madison High School, Madison, MS 39110; d Department of Chemistry, Physics and Atmospheric Science, Jackson State University, Jackson, MS 39217

**Keywords:** Visualization, Uranium, Vertical Transport, Pollution, Arid Landscape

## Abstract

Uranium (U) is a naturally occurring, radioactive, toxic trace element that poses severe risks to public and environmental health. Depleted uranium (DU) is widely used in military munitions, including penetrators. Our previous studies showed that in arid landscapes, water-soluble U released from corroded DU penetrators that were buried underground were co-transported upwards with water by evaporation-driven capillary action and eventually precipitated on the ground surface. The first objective of this study was to develop a visualization system to simulate this complex U upward transport mechanism involving cyclic capillary wetting-drying cycles. Multiple visual components such as visual elements, canvases, and animations were created using JavaScript, HTML, and CSS programming languages and coordinated to visualize this biogeochemical process in arid ecosystem landscapes. The second objective was to develop an interactive visualization exercise to allow users to study the effect of the type of capillarity solutions on the speed of the U upward transport. This study is significant in the following aspects:

• Contributing a clear and comprehensible visualization of the complex U transport mechanism;
• Developing a novel visualization coding framework with more advantages in simulating heavy metal upward transport mechanisms than regular software-based simulations; and
• Providing educational uses such as an instructional tool in secondary and college STEM classrooms, an outreach material in promoting student interest in STEM topics and raising public awareness of U pollution, and an educational aid for understanding U mobility in order to develop effective heavy metal pollution control and remediation strategies and policies.

## Graphical Abstract

**Figure:**
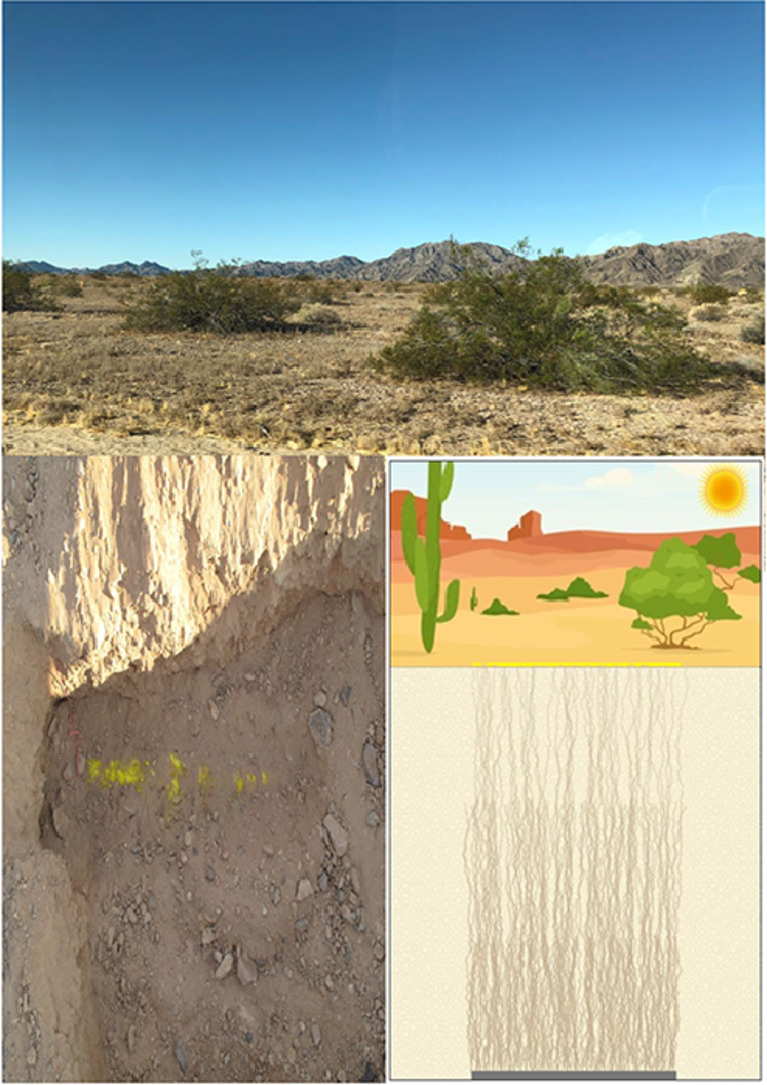

Specifications Table

**Table:** 

Subject Area:	Environmental Science
More specific subject area:	Heavy metal pollution control and remediation
Method name:	Developing a Novel Computer Visualization System to Simulate the Uranium Upward Transport Mechanism: Uranium Pollution in Arid Landscapes
Name and reference of original method:	J.A. Kazery, R. Yang, L. Bao, Q. Zhang, M. James, S. Dasari, F. Guo, J. Nie, S.L. Larson, J.H. Ballard, H.M. Knotek-Smith, R. Unz, P. B. Tchounwou, F.X. Han, Horizontal and Vertical Transport of Uranium in an Arid Weapon Tested Ecosystem, *ACS Earth and Space Chemistry* 6 (5) (2022) 1321–1330. https://doi.org/10.1021/acsearthspacechem.2c00028.
Resource availability:	N/A

## Method details

### Background

Uranium (U) is a naturally occurring toxic trace element and radioactive contaminant in the environment. The natural abundance of U is in a range of 0.3-11.7 mg kg^−1^ in rocks and soils, with an average of 3 mg kg^−1^ [1,2]. Anthropogenic activities such as industrialization, mining and processing, nuclear manufacture, power plants, and the use of depleted uranium products all result in the release of U into the environment. Three main U isotopes (U-238, U-235, and U-234) are all radioactive.

Depleted uranium (DU) is the by-product of the U enrichment process. DU has a lower content of the fissile isotope U-235, thus less radioactive than natural U. Due to its pyrophoric properties and high density, DU has uses in military applications. DU is widely used in ballasts in aircraft, bullet heads in military munitions, including penetrators, and radiation shields in medical equipment. During military conflicts over the past 30 years, more than a thousand tons of DU has been used, resulting in unknown amounts of DU metal fragments deposited on battlefields and army testing sites [3], [4], [5], [6], [7], [8].

DU poses multiple health risks due to its radiological and chemical toxicity. Because DU is a low radioactive α-emitter, its acute risk is not likely from external exposure. However, its potential hazard results from internal exposure, especially considering that DU particulates are small enough for inhalation [9], [10]. Uranium from embedded DU fragments may also be exposed and redistributed, resulting in high levels of several oncogenes associated with carcinogenesis [9], [10].

There have been several studies regarding the transport and contamination of U in various environments [7], [8],11]. We previously reported that significant amounts of U had been accumulated in weapon-tested sites such as the Yuma Proving Ground site [5], [6], [7], [8]. Both horizontal and vertical types of transport of U were observed at the site. The horizontal transport was mainly driven by high surface water runoff that could significantly reduce the concentrations of U on the ground as close as 20 meters away from the DU source [7,8]. In a desert landscape, U released from corroded DU penetrators that were buried underground went through vertical transport and eventually precipitated on the surface [7], [8]. Plant absorption of U through the roots from polluted soils and animal consumption through the food chain also played essential roles in transporting U throughout the ecosystem [8].

Kazery et al. (2022) proposed a mechanism of upward transport of U in arid conditions, which was driven by water movement including both leaching with gravity (downward movement) and evaporation (upward movement) [8]. During the wetting stage (e.g., during the rain) of the wetting-drying cycle of water in soils, water leached in soils due to gravity, and soluble U was dissolved in water in the soil pore; during the drying stage of the cycle, the dissolved U such as uranyl (UO_2_^2+^) was co-transported with water upwards due to evaporation-driven capillary action [8]. With multiple wetting-drying cycles, dissolved U was transported toward the surface and precipitated as yellow-colored uranium oxides [8]. Such a U vertical transport mechanism through alternating wetting and drying conditions has been tested and confirmed under laboratory-controlled conditions [8,12].

The objectives of this study were as follows:

(a) To develop a computer visualization system to simulate this complex upward transport mechanism of U involving cyclic wetting-drying cycles;
(b) To create an interactive visualization exercise to allow users to study the effect of the type of capillarity solutions (i.e., pore solutions in arid soils) on the speed of the U upward transport.

### Tools used

This study created a web-based visualization using JavaScript, HTML, and CSS programming languages. HTML and CSS were used to provide instructions for the web browser to display a variety of components such as text, images, and forms on the screen and define visual elements inside a webpage, including their colors, sizes, and positions. JavaScript was used to support complex logic and create animation effects inside the web browser by programming gradual changes in the visual elements. Tools used in the study included Sublime Text (a generic editor software) and a Chrome web browser.

### Procedures

The procedures in this study included designing and creating multiple components, including visual elements (e.g., the Sun and rain), designated visualization areas called canvases (e.g., the sky, soil, and water canvases), and animations (e.g., the raining, wetting, drying, and transporting animations). These components were coordinated to visualize a complete U upward transport mechanism in arid landscapes.

#### Visual elements

The following elements were created and embedded in the visual design of the visualization:

a) The Sun and rain

The Sun and rain elements were used to simulate arid landscapes’ natural drying and wetting processes. When it rained during the wetting stage of the wetting-drying cycle, raindrops fell from the sky, accumulated on the ground, and then penetrated underground. When the rain stopped and the Sun came out, water in the soil pores gradually evaporated during the drying stage of the cycle.

b) Uranium block

**Fig. 1:**
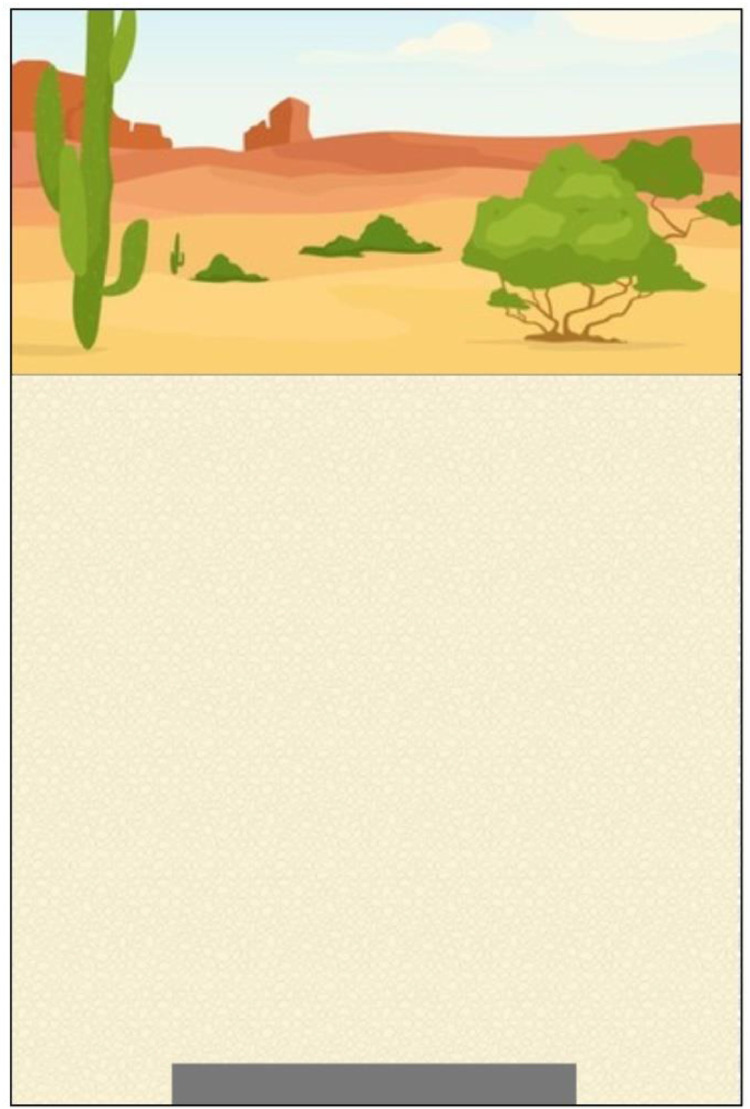
Initial simulation screen with visual elements including the arid landscape, underground soil profile, and buried uranium block.

This uranium block element represented the DU penetrator that was buried underground (see the black block in Fig. 1). Over time, the surface of the DU penetrator was corroded, and U from the corroded parts was dissolved in liquid water and transported upwards. Gradually, the penetrator lost its mass, becoming smaller with every wetting-drying cycle.

c) Soil profile

The soil area represented the underground soil or sand where water leached through during the wetting stage and evaporated during the drying stage (see the light brown area under the ground in Fig. 1). Soil attributes, such as the pore sizes and the type of capillarity solutions in the soil pores, affected the capillary effect, resulting in U moving upwards through the soil profile.

#### Canvases

**Fig. 2:**
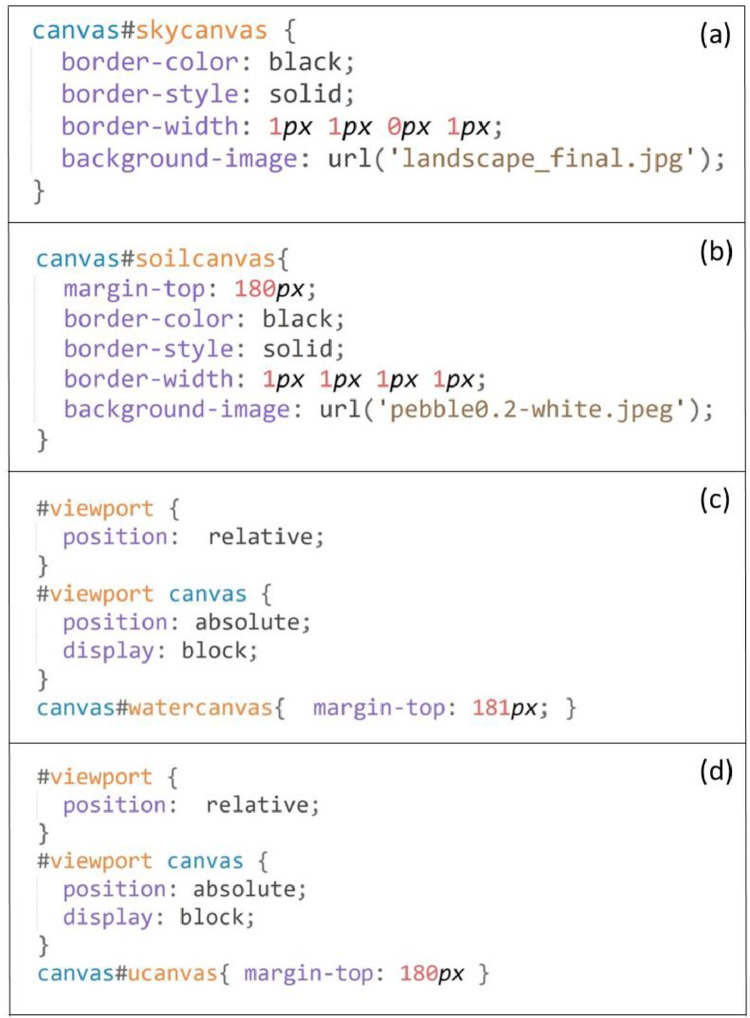
Code snippets for visual definitions of the canvases (a-sky canvas; b-soil canvas; c-water canvas; d-uranium canvas).

In HTML, a canvas is a designated visualization area with a fixed height, width, and position, inside which many colors and shapes could be drawn using JavaScript. This simulation was developed on an area of a 360 pixels (width) by 540 pixels (height) rectangle. Four independent canvases, the sky, soil, water, and uranium canvases, were created in this area, next to or overlapping with each other (refer to the code snippets in Fig. 2). Multiple canvases were placed on each other to create rich visual effects.

a) Sky Canvas

A 360 × 180 pixel sky canvas was developed and placed on top of the simulation area (see Fig. 1). An arid ecosystem landscape picture was used as the background for this canvas. When it rained, an animation featured called “raining” (to be described later) drew falling raindrops for a specified time. When the rain stopped, a picture of the Sun was added to the top right corner, simulating the drying stage of the wetting-drying cycle.

b) Soil Canvas

A 360 × 360 pixel soil canvas was added right below the sky canvas (see Fig. 1). It represented the underground soil profile. An image of light brown colored soils was used as the background for this canvas. At the bottom of the soil canvas, a rectangle black block was added to represent the uranium block. An animation feature called “mass-reducing” was developed to animate the process of mass loss of the uranium block.

c) Water Canvas

A 360 × 360 pixel water canvas was developed, placed below the sky canvas, and overlapped with the soil canvas (see Fig. 1). The water canvas did not need any background. Two animation features called “wetting” and “drying” were developed to simulate the processes of water leaching (downward movement) and evaporation (upward movement), respectively, on this canvas.

d) Uranium Canvas

A 360 × 360 pixel uranium canvas was developed to overlap with the soil canvas and water canvas (see Fig. 1). An animation feature called “transporting” was added to the uranium canvas to simulate the U upward movement in soils.

#### Animations

JavaScript was used in this study to draw strokes and shapes with an array of colors, sizes, and positions on multiple canvases. Another important JavaScript feature used was time intervals, which allowed a JavaScript function to be executed repeatedly with a fixed time (interval) between each execution. Animation effects of a moving subject were created by repeatedly drawing the subject on a canvas at changed positions along the moving course. The following animations were created:

1) Raining Animation

**Fig. 3:**
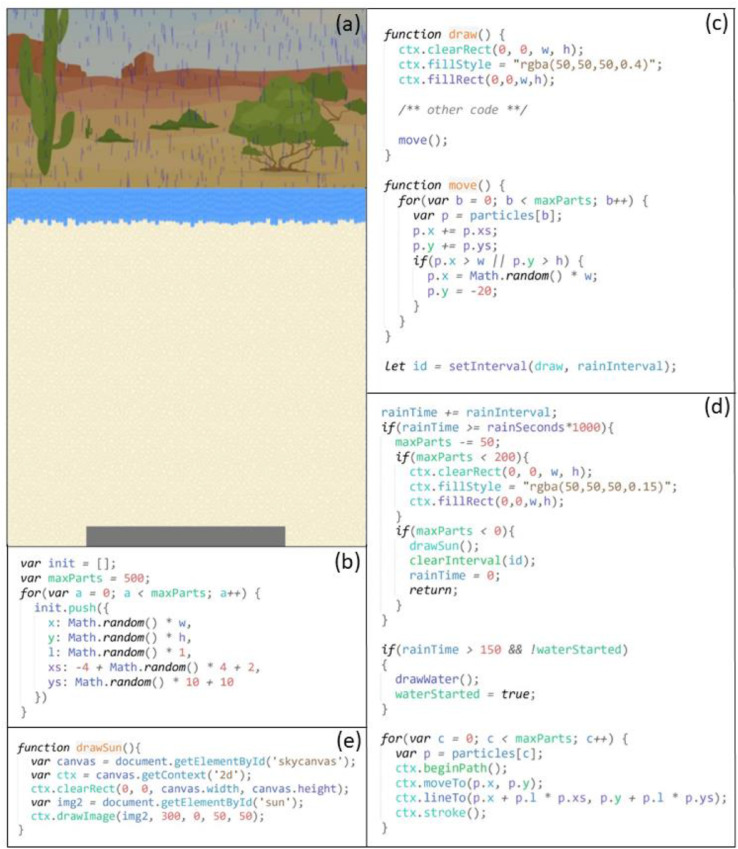
Screenshot (a) and code snippets for the raining animation (b-defining the initial positions of the 500 raindrops; c-falling action of the raindrops; d-rain stopping gradually; e-the Sun appearing in the top right corner of the sky canvas)

The raining animation was developed on the sky canvas (see Fig. 3). When it started to rain, a gray layer with 40% opacity was drawn over the entire canvas to simulate the dark sky during the rain. In addition, 500 short vertical strokes were drawn in random positions, simulating the falling raindrops.

In simulating the falling action of the raindrops, previous vertical strokes were erased every 0.1 seconds and redrawn in a lower position but shifted slightly and randomly to the left or the right. When a raindrop reached the ground or shifted outside the canvas area, it was moved back to the top of the sky at a random position to continue the falling process. Together, they created a visual effect of raindrops falling from the sky continuously.

After it rained for a few seconds, 50 raindrops were removed every 0.1 seconds to simulate the rain stopping gradually. When no raindrops were left on the screen, the gray opaque layer was removed to fully expose the original landscape background picture. At the same time, a picture of the Sun was drawn in the top right corner to indicate the start of the drying stage of the wetting-drying cycle. The raining animation thus stopped.

2) Wetting Animation

**Fig. 4:**
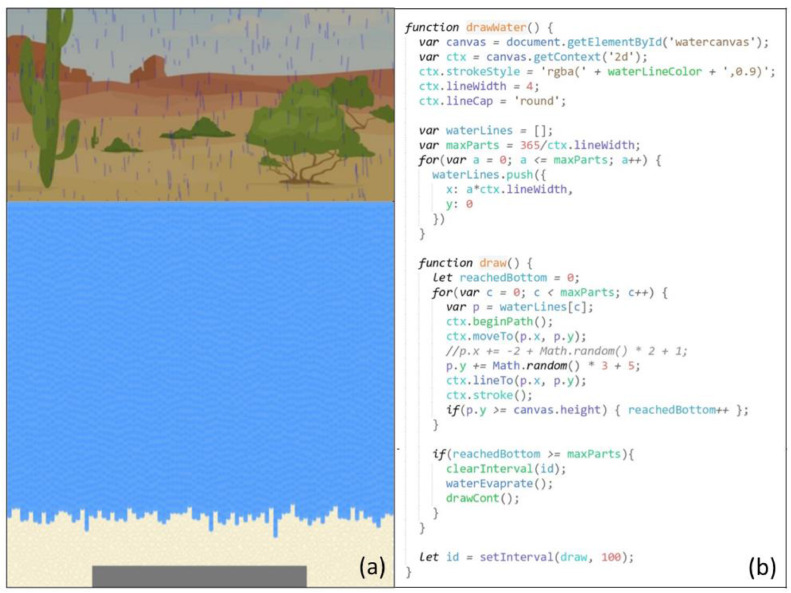
Screenshot (a) and code snippet (b) for the wetting animation during the wetting stage of the wetting-drying cycle, simulating water leaching through soils during the rain.

The wetting animation was created to simulate water leaching through soils when it was raining during the wetting stage of the wetting-drying cycle (Fig. 4). It was developed on the water canvas, which was below the sky canvas, and started at the same time as the raining animation. Many short blue lines representing leaching water streams were initially drawn on the top of the canvas, right below the ground line. For every 0.1 seconds, the blue lines were extended for random lengths between 5 and 8 pixels, simulating the water stream leaching effect. When all the blue lines reached the bottom, the entire water canvas was colored blue with 100% opacity, demonstrating soil pores being fully saturated with water. The above rain animation also stopped at this time, and the Sun appeared.

3) Drying Animation

**Fig. 5:**
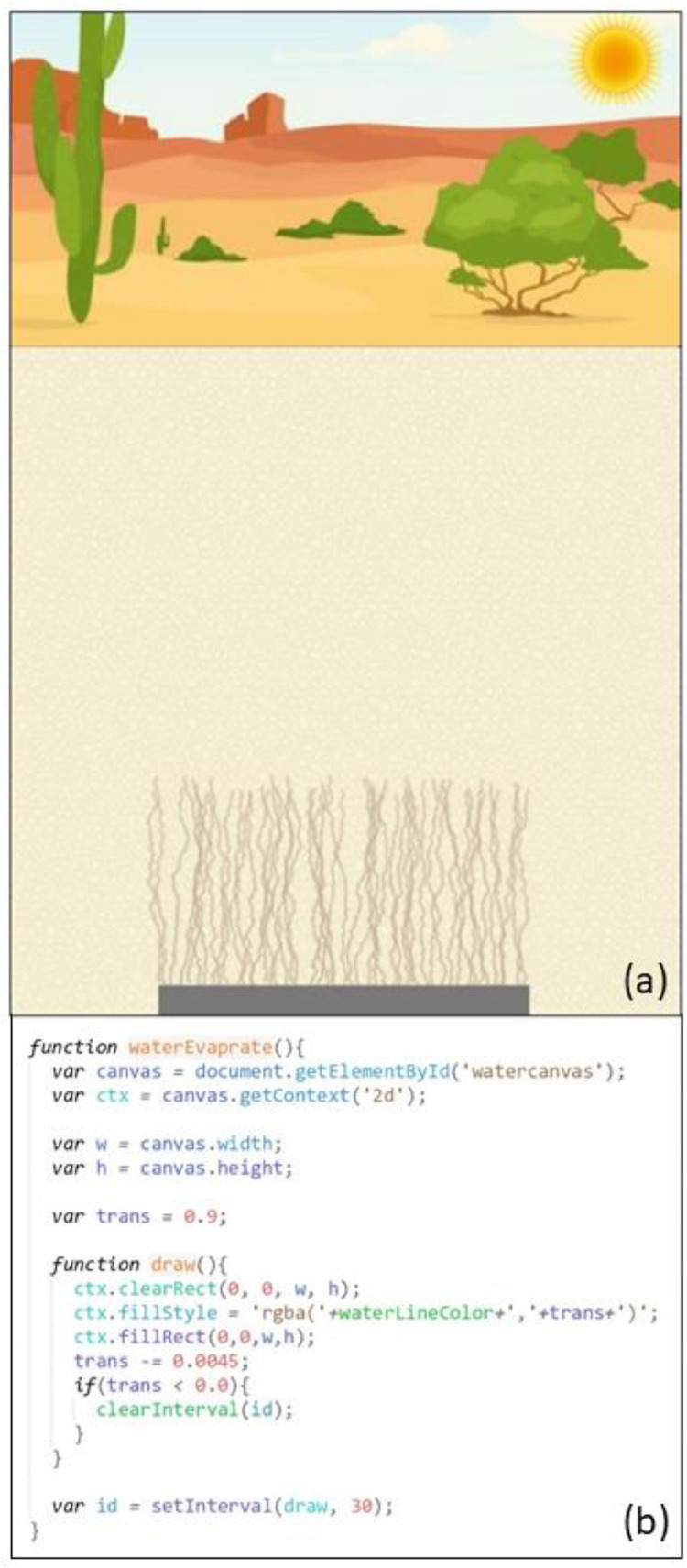
Screenshot (a) and code snippet (b) for the drying animation during the drying stage of the wetting-drying cycle, simulating soil pore water evaporation. The U upward transport happened simultaneously and was controlled by the transporting animation.

The drying animation was developed to simulate the evaporation process of soil pore water during the drying stage of the wetting-drying cycle, resulting in the U upward transport through evaporation-driven capillary action (see Fig. 5). When the previous wetting animation stopped, the opacity of the blue area was 100%. During the drying animation, the opacity was gradually reduced every 0.03 seconds, simulating the drying or evaporation of water. The drying animation stopped when the blue area became transparent (0% opacity), meaning that water had entirely evaporated.

4) Transporting Animation

**Fig. 6:**
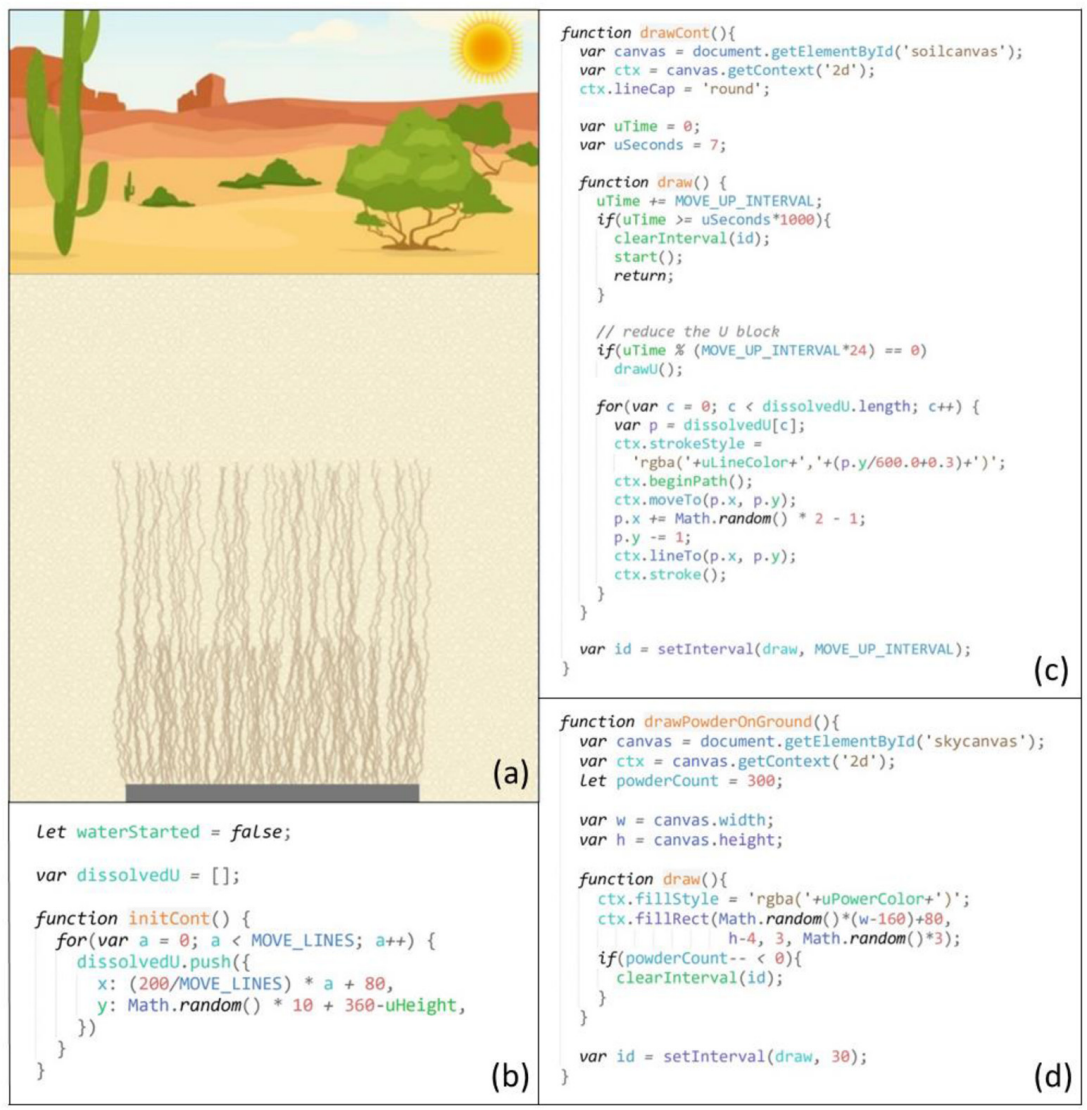
Screenshot (a) and code snippets for the transporting animation, simulating U moving up through multiple wetting-drying cycles (b-defining the initial positions of U upward movement tracing lines; c-U upward movement; d-U surfacing on the ground)

The upward transporting animation was developed on the uranium canvas (see Fig. 6), which overlapped with the water canvas. It started from the uranium block drawn at the bottom of the water canvas and at the same time as the drying animation. During the transporting animation, dissolved U from the corroded parts of the uranium block moved upwards in soils through evaporation-driven capillary action.

**Fig. 7:**
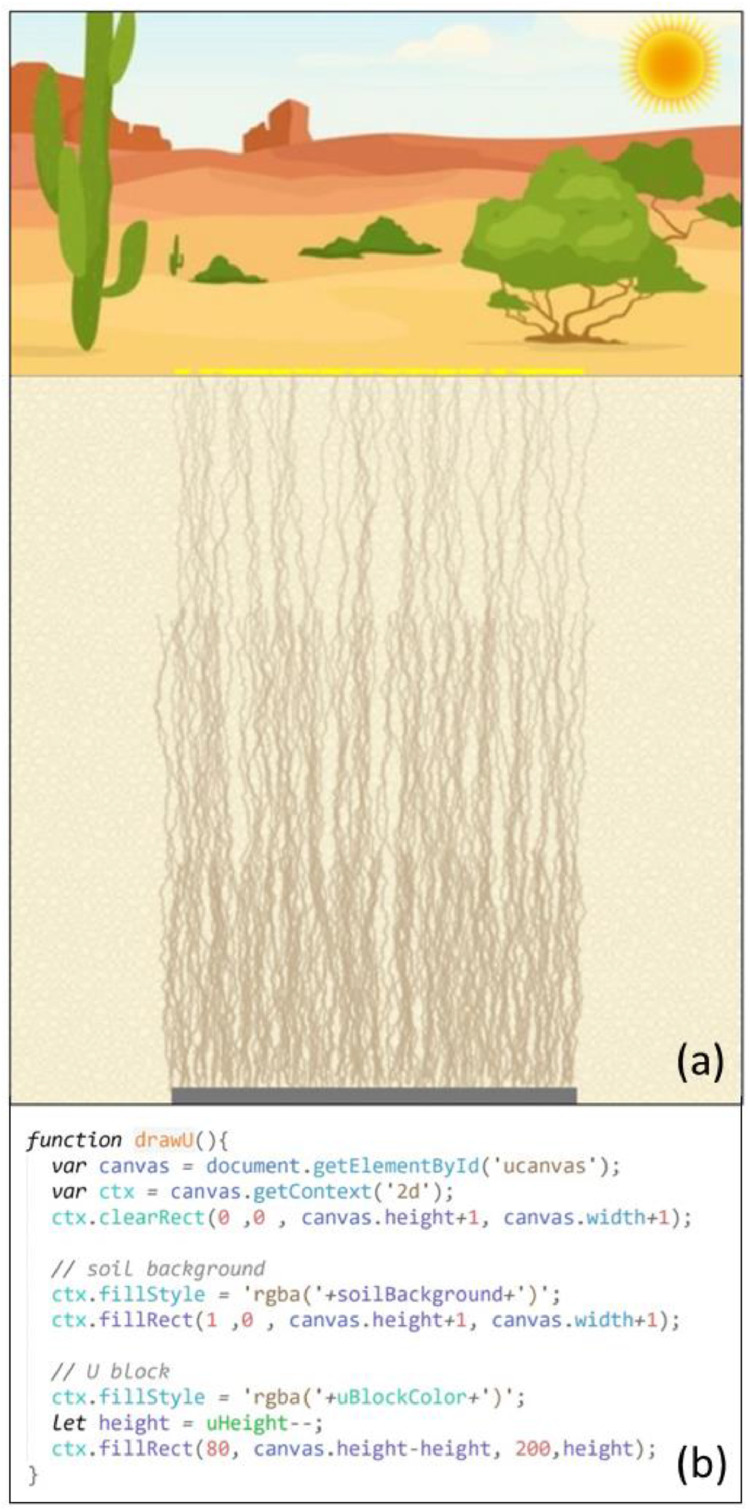
Screenshot (a) and code snippet (b) for the mass-reducing animation, simulating the underground U block losing mass gradually. Screenshot (a) also marked the final stage of the U upward transport mechanism when the U frontline reached the surface (note the yellow-colored uranium pollutants deposited on the ground), which was controlled by the transporting animation.

The traces left by U when they moved up were demonstrated by small brown lines. All the brown lines were drawn to start from the uranium block. To simulate the U upward movement, the lengths of the lines increased by one or two pixels every 0.06 seconds and shifted slightly and randomly to the left or the right. After U reached the surface, yellow dots were drawn on the ground to simulate the precipitation of U pollutants on the surface (Fig. 6d and Fig. 7a).

5) Mass-Reducing Animation

The mass-reducing animation simulated the mass loss of the uranium block while dissolved U species from the corroded uranium block were transported upwards (see Fig. 7). In each wetting-drying cycle, the uranium block was redrawn with a lower height than the previous block drawing. The mass-reducing and transporting animations were coordinated to simulate the U upward movement at the expense of mass loss of the uranium block through multiple wetting-drying cycles.

#### Animation coordination

All the animations were put together in a coordinated fashion to visualize a complete U upward transport mechanism in arid landscapes. Specifically, the raining animation and the wetting animation started simultaneously. When the raining animation stopped, the wetting animation stopped, and the drying animation started. At the same time, the transporting and mass-reducing animations started. After the evaporation process of the drying animation ended, the transporting and mass-reducing animations also stopped because U could no longer move up when soils were entirely dried, marking the end of the first wetting-drying cycle.

Then, another raining animation started, and the above wetting-drying cycle repeated. In the previous cycle, some U was transported upwards but had not yet reached the surface when soils became dried entirely. During the wetting stage of a new cycle, U was dissolved by more leaching water that was replenished from the new rain and then continued to be co-transported upwards with water through evaporation-driven capillary action during the drying stage. New U from the buried uranium block was also dissolved and moved up together with water, increasing the total amount of U transported upwards through soils.

The wetting-drying cycles repeated until the frontline of U reached the surface. In this visualization, it was set that it would take U three wetting-drying cycles to arrive at the surface and deposit as yellow-colored uranium minerals such as uranium oxides on the ground [8,12] (Fig. 7).

### Validation with interactive visualization

To verify and expand the usefulness of the visualization system in engaging active learning and deeper understanding, an interactive visualization exercise was developed to allow users to study the effect of the type of capillarity solutions (i.e., pore solutions in arid soils) on the speed of the U upward transport. As stated earlier, soil attributes such as the type of capillarity solutions affected capillary action and therefore impacted the speed of U movement in soils [8,12].

**Fig. 8:**
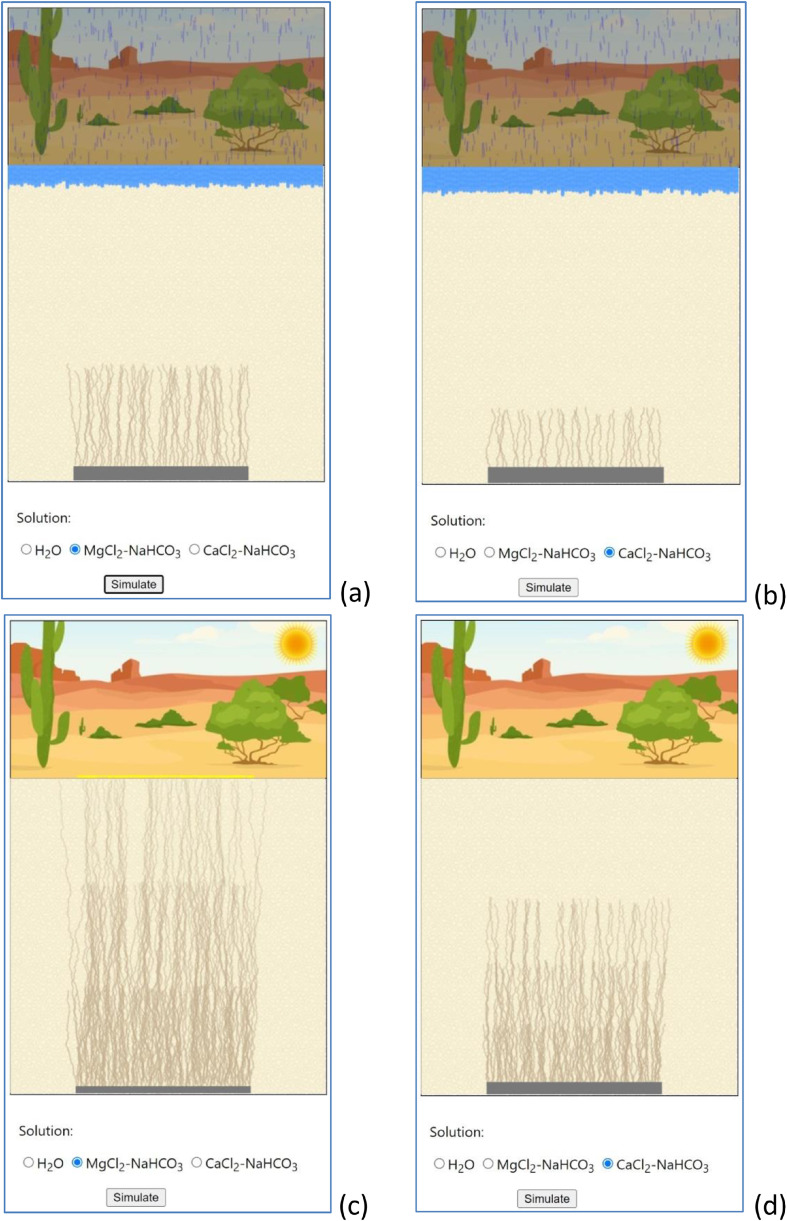
Interactive visualization to allow users to study the effect of the type of capillarity solutions on the speed of the U upward transport (a-U in MgCl_2_-NaHCO_3_ at Time 1; b-U in CaCl_2_-NaHCO_3_ at Time 1; c-U in MgCl_2_-NaHCO_3_ at Time 2; d-U in CaCl_2_-NaHCO_3_ at Time 2).

Based on the wet lab results [8,12], an interactive visualization exercise was developed to simulate the impact of the type of capillarity solutions. An interactive selection of capillarity solutions was provided. The upward movement speed would change based on the user's actual selection of the solution. As exemplified in Fig. 8, the transport distance of U in MgCl_2_-NaHCO_3_ was different from those in CaCl_2_-NaHCO_3_ within a given time. As a result, while it may take only n wetting-drying cycles for U to reach the surface in MgCl_2_-NaHCO_3_, it may take m wetting-drying cycles for U in CaCl_2_-NaHCO_3_. By changing the pore solutions and interacting with the simulation exercise repetitively, users would gain a deeper understanding of the U upward transport mechanism and the potential effects of soil attribute variables on the mobility of U.

## Final remarks

In this study, a novel web-based visualization system has been developed to visually demonstrate the U upward transport mechanism in arid landscapes. The computer visualization makes the complex process easy to understand regarding how depleted uranium (DU) released from corroded DU penetrators is transported upwards by evaporation-driven capillary action and eventually deposited on the surface through multiple wetting-drying cycles. Users can also interact with the system by choosing various pore solutions to investigate the effect of capillarity solutions on the speed of the U upward transport. The interactive visualization appears more appealing to youth (e.g., high school students) for educational purposes than a pure textual explanation of the process.

This study is significant not only in contributing a clear and comprehensible visualization of the complex U transport mechanism but also in developing a novel visualization coding framework that has more advantages in simulating heavy metal upward transport mechanisms than regular software-based simulations. The programming-based method developed in this study is not limited by the capability of any existing simulation software. As a result, it provides more flexibility, has more refined controls on simulation details, and supports wider varieties of user interactions than software-based simulations.

The interactive visualization system developed in this study also provides educational uses such as an instructional tool in secondary and college STEM classrooms, an outreach material in promoting student interest in STEM topics and raising public awareness of U pollution. Also it provides an educational aid for understanding U mobility in order to develop effective heavy metal pollution control and remediation strategies and policies.

## Declaration of Interests

The authors declare that they have no known competing financial interests or personal relationships that could have appeared to influence the work reported in this paper.

## Data Availability

Data will be made available on request.
